# The evolution of COVID-19 vaccine hesitancy in Sub-Saharan Africa: evidence from panel survey data

**DOI:** 10.1186/s12919-023-00266-x

**Published:** 2023-07-06

**Authors:** Philip Wollburg, Yannick Markhof, Shelton Kanyanda, Alberto Zezza

**Affiliations:** 1 World Bank, Development Economics Data Group, Washington, DC USA; 2 UNU-MERIT, United Nations University, Maastricht, Netherlands; 3 National Statistical Office, Zomba, Malawi

**Keywords:** COVID-19, Vaccination, Africa, Vaccine hesitancy

## Abstract

**Background:**

COVID-19 vaccination efforts are lagging in Sub-Saharan Africa, as just over 20 percent of the population has been fully vaccinated. COVID-19 vaccine hesitancy is considered important as a prerequisite for widespread vaccine take-up. Here, we study the dynamics of vaccine acceptance, its correlates, and reasons for hesitancy over time, drawing on two years of panel survey data.

**Methods:**

In this observational study, we use multiple rounds of data from national High Frequency Phone Surveys (HFPS) in five countries in East and West Africa (Burkina Faso, Ethiopia, Malawi, Nigeria, and Uganda), covering a period between 2020 and 2022. The surveys are cross-country comparable and draw their samples from nationally representative sampling frames. Based on this data source, the study presents population-weighted means and performs multivariate regression analysis.

**Results:**

COVID-19 vaccine acceptance was high throughout the study period (68% to 98%). However, acceptance levels were lower in 2022 than in 2020 in three countries (Burkina Faso, Malawi, Nigeria), and higher in one country (Uganda). Moreover, individuals are observed to change their stated vaccine attitudes between survey rounds, to a limited extent in some countries (Ethiopia) and more frequently in others (Burkina Faso, Malawi, Nigeria, Uganda). Vaccine hesitancy is higher in richer households, and those residing in urban areas; among women and those better educated. Hesitancy is lower in larger households and among heads of the household. The main reasons for hesitancy are concerns about side effects of the vaccine, its safety and efficacy, as well as assessments of COVID-19 risk, though these reasons fluctuate over time.

**Conclusions:**

Reported COVID-19 vaccine acceptance levels remain far above vaccination rates in the study countries, suggesting that vaccine hesitancy is not the primary obstacle to reaching greater vaccine coverage, which may instead be related to access and delivery barriers as well as supply shortages. Nevertheless, vaccine attitudes appear malleable so that continued efforts are needed to retain high levels of vaccine acceptance.

**Supplementary Information:**

The online version contains supplementary material available at 10.1186/s12919-023-00266-x.

## Background

On December 8, 2020, the first COVID-19 vaccine outside a clinical trial was administered [1]. Two years onwards, the COVID-19 vaccine drive has become the largest vaccination effort in history and has reached over 5.4 billion people globally [2, 3].

Challenges for COVID-19 vaccination campaigns differ from past vaccination efforts. Instead of taking years or even decades of development, the first COVID-19 vaccines achieved emergency approval within less than a year of the first recorded cases of the disease [4, 5]. Such rapid development has given rise to concerns among members of the public regarding the safety of COVID-19 vaccines and has led to additional scrutiny [6]. Following the availability of COVID-19 vaccines, the severity of the pandemic that ensued over the course of 2020 has also put time pressure on the rollout of vaccination campaigns, has led to supply shortages and resulted in vastly heterogeneous availability of COVID-19 vaccines across the globe [7]. Consequently, many low- and middle-income countries that lack the ability to produce vaccines of their own have relied on donations of vaccine doses from vaccine-producing countries and face infrastructural challenges in the rollout of their vaccination campaigns. Some of these challenges have still not been completely overcome 2 years after [8].

A region that has been of particular concern in the quest for global COVID-19 vaccine coverage has been Sub-Saharan Africa. Sub-Saharan Africa is the world’s poorest region where the pandemic impinged on already vulnerable livelihoods [9–12]. In addition, capacity to develop or procure COVID-19 vaccines is lowest in the region and structural barriers that complicate vaccine delivery are pervasive [8, 13–15]. As a result, COVID-19 vaccine coverage in Sub-Saharan Africa is trailing other regions [16]. Furthermore, Sub-Saharan Africa is a data-scarce environment with typically little robust information on issues such as vaccine acceptance or barriers of access that can inform the rollout of vaccination campaigns [17]. Yet, Sub-Saharan Africa is home to over 1.16 billion people, about 15% of the world’s population [18]. This makes it a systemically important region in the global effort to contain COVID-19 and end the pandemic [19].

The special status of COVID-19 vaccination campaigns in Sub-Saharan Africa have created the need for research to fill knowledge gaps around the effective mass delivery of COVID-19 vaccinations in the region [8, 16, 20–23]. In an effort to fill this gap, the World Bank supported a series of cross-country comparable high-frequency phone surveys (HFPS) in early 2020 that collect recurring information on vaccine hesitancy, uptake, barriers of access and information transmission in the context of COVID-19 vaccines in Sub-Saharan Africa.

In this article, we bring together two years of data and findings from the HFPS across five Sub-Saharan African countries. We focus on COVID-19 vaccine acceptance as a prerequisite for widespread vaccine coverage in the region and a matter of particular concern given the special circumstances of COVID-19 vaccine development [24]. COVID-19 vaccine hesitancy has been the subject of a number of studies across low- and middle-income countries, including in Sub-Saharan Africa [16, 20, 21, 24–30]. These studies suggest generally high levels of vaccine acceptance in Sub-Saharan Africa however, there is a lack of longitudinal evidence. Such longitudinal evidence is needed to understand the dynamics of vaccine acceptance in the region with some of the world’s lowest COVID-19 vaccine coverage figures. By virtue of the recurring nature of the HFPS, we are able to study vaccine attitudes and their correlates at the country-level and among a panel of individuals over a 2-year time horizon.

The evidence we present supports the notion that vaccine hesitancy has not been the binding constraint to reaching high levels of COVID-19 vaccine coverage at any point of the pandemic. As such, it complements recent calls to focus on the removal of structural and supply-side barriers for COVID-19 vaccination in low-income countries in general and Sub-Saharan Africa in particular [8, 16, 31]. It also resonates with calls for more equitable sharing of vaccine doses and proposals to develop domestic manufacturing capacities [7, 13–15, 19, 23].

## Methods

### Data

We use data from High Frequency Phone Surveys (HFPS) in five countries in Sub-Saharan Africa: Burkina Faso, Ethiopia, Malawi, Nigeria, and Uganda. The surveys were conducted by study countries’ national statistical organizations (NSOs), supported by the World Bank’s Living Standard Measurement Study (LSMS). Since May 2020, the LSMS-supported HFPS have collected cross-country comparable longitudinal data on a wide range of topics, focused on COVID-19 impacts on households and individuals. The choice of countries was based on the existence of a pre-COVID-19 in-person survey infrastructure, including contact information of survey respondents who had previously been interviewed. An existing survey infrastructure made possible drawing a high-quality sample and the fast deployment of the surveys [32].

Vaccine hesitancy related information was collected in 2020, 2021, and recently in 2022, depending on the study country. This study uses five rounds of data for Nigeria (Oct 2020, Feb 2021, Dec 2021-Jan 2022, Mar-Apr 2022, Jul-Aug 2022), four rounds for Malawi (Oct-Nov 2020, Apr 2021, Feb 2022, Jul-Sep 2022), Uganda (Oct-Nov 2020, Feb 2021, Sep-Nov 2021, Aug-Sep 2022) and Burkina Faso (Dec 2020, May-Jun 2021, Apr-May 2022, Aug-Sep 2022), and two rounds of data for Ethiopia (Sep-Oct 2020, Feb 2021).

### Sampling and sample representativeness

The HFPS target population is a national sample of households, with one adult main respondent per household, selected purposively to be knowledgeable of the affairs of the household and its members. The initial sampling frames of the HFPS are based on contact lists of households visited during recent, pre-pandemic, nationally representative face-to-face surveys of the LSMS-Integrated Surveys on Agriculture (LSMS-ISA) series. The LSMS-ISA surveys employ a two-stage stratified cluster sampling approach. Those face-to-face households for which a contact phone number had been collected in the most recent visit before the pandemic were included in the HFPS sampling frames, and either all households or a random subsample of households were selected to be interviewed as part of the HFPS.

In the low- and middle-income settings at hand, mobile phone coverage is not universal, meaning that some households cannot be reached over the phone. Therefore, the sample selection of phone surveys may not yield samples fully representative of the general population. Phone surveys also experience an extent (albeit limited) of non-response and attrition. The HFPS therefore use recalibrated sampling weights (based on propensity score and post-stratification methods), which mitigate sample selection biases [33–35]. In each household, one main respondent over the age of 15 was interviewed, selected to be knowledgeable of the household and its members. This purposive selection has been found to overrepresent certain population groups [35]. The sample sizes for each country are shown in Additional file 1.

### Survey instrument and variables

To capture vaccine hesitancy and its reasons, survey instruments were developed and harmonized across countries while allowing for a degree of contextualization where necessary. The survey instruments consisted of a series of questions to the main respondent: (i) To gauge willingness, respondents were asked whether they were willing or planning to be vaccinated against COVID-19. Vaccine hesitancy was defined based on this question if the answer was ‘no’ or ‘not sure’. (ii) In these cases, a follow-up question on the reasons for hesitancy was asked. In the survey rounds conducted before COVID-19 vaccines were available, vaccine acceptance questions were posed in the hypothetical, asking respondents if they would be willing to get vaccinated if an approved COVID-19 vaccine was available. When vaccines became more widely available, questions were changed to reflect this development, asking respondents if they were planning to be vaccinated. Respondent and household characteristics used in parts of the ensuing analysis were drawn from information collected in the pre-pandemic survey visit or other questionnaire modules administered in the HFPS.

### Estimation methods

Reported estimates are population-weighted means with their 95% confidence intervals where applicable, computed using the recalibrated phone survey weights. To explore how individual and household characteristics correlate with the willingness to get vaccinated, we make use of a multivariate logit regression setup, again using recalibrated phone survey weights.

## Results

### Vaccine acceptance over time

**Fig. 1:**
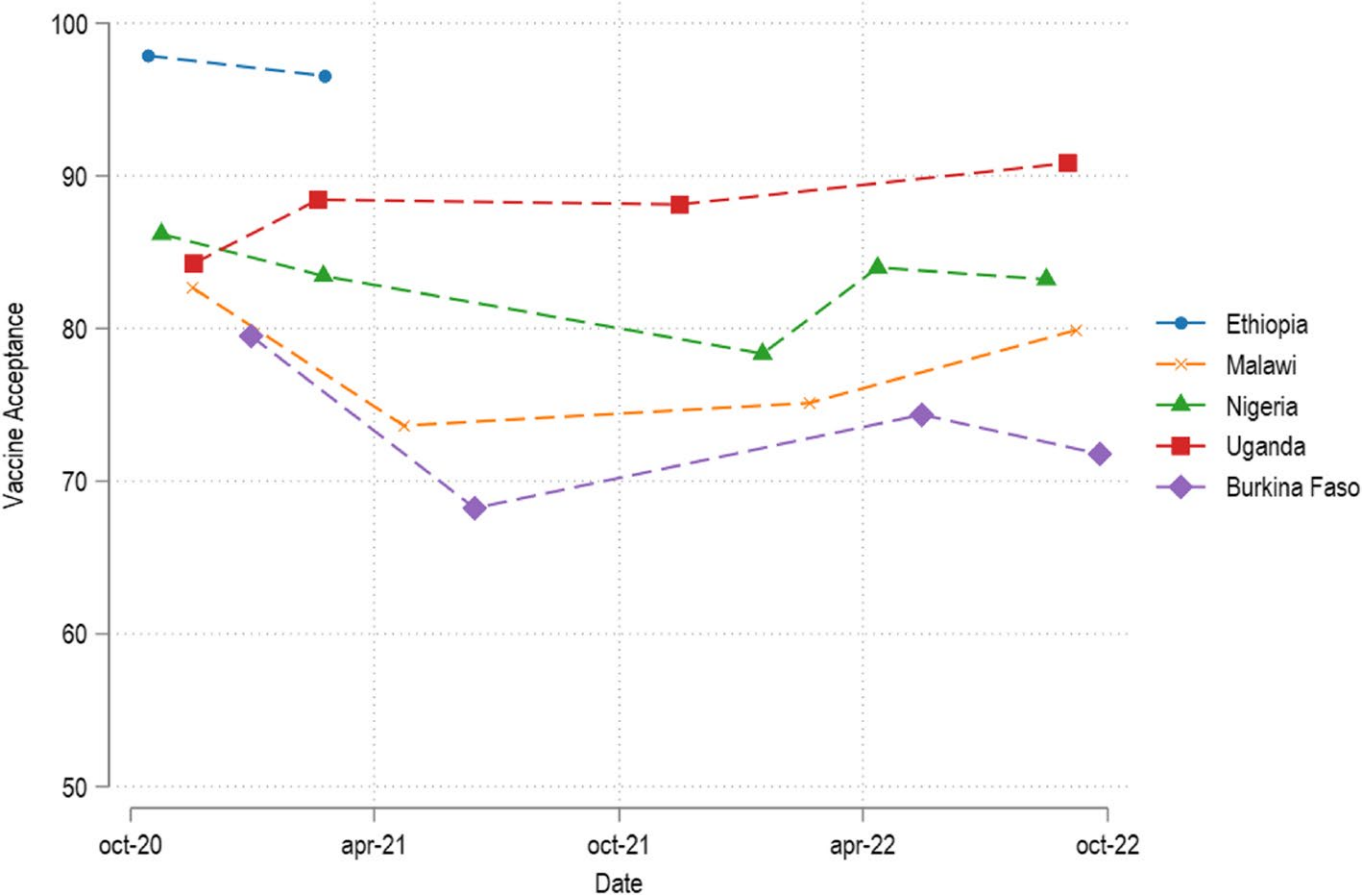
Vaccine acceptance over time

We show reported vaccine acceptance rates in our study countries in 2020, 2021, and 2022 in Fig. 1. Broadly speaking, we find sustainedly high levels of vaccine acceptance, remaining well above 70% in almost all cases. However, there is considerable country and time heterogeneity. In all countries but Uganda, we observe vaccine acceptance rates to initially decline over the first year of data collection. In Ethiopia, support remains nevertheless very high, starting at 97.9% in 2020 and declining to 96.5% in 2021. In Nigeria, there is at first a stronger decline from 86.2% in 2020 to 83.4% in 2021 to 78.4% in early 2022 with a subsequent recovery back to 84.0% in April and 83.2% in August 2022. In Malawi and Burkina Faso, there are steeper declines in acceptance from 2020 to 2021 (Malawi: from 82.7% in 2020 to 73.6% in 2021; Burkina Faso: 79.5% in 2020 to 68.2% in 2021) – but acceptance rates rebounded in 2022, to levels slightly below the initial rates of 2020 in the case of Malawi (79.9%) and somewhat further below initial rates in Burkina Faso (71.8%). Finally, in Uganda, acceptance rates have increased overall between 2020 and 2022 (from 84.3% in 2020 to 90.8% in 2022).

### Correlates of vaccine acceptance

**Fig. 2:**
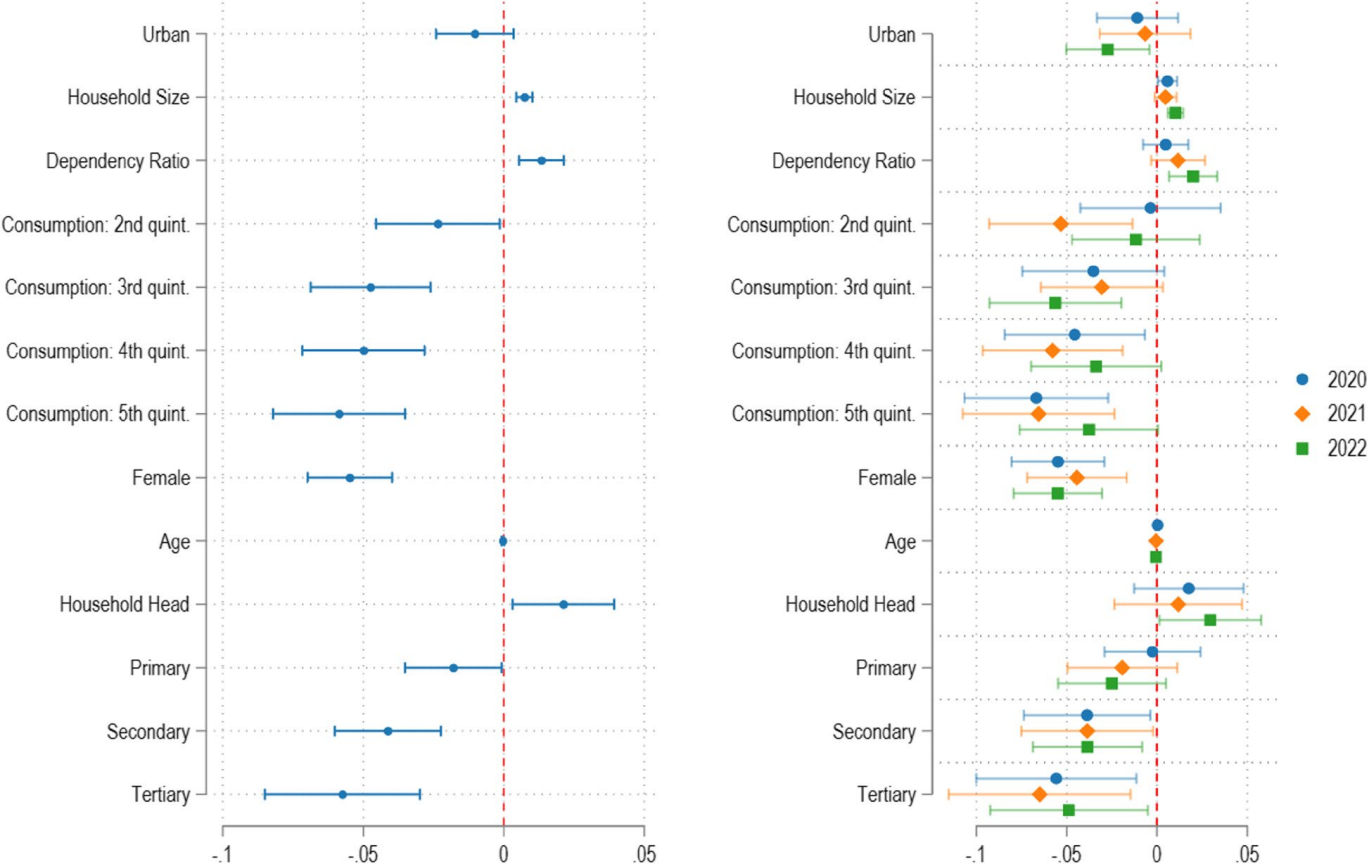
Correlates of vaccine acceptance across countries and across years

There are some differences in COVID-19 vaccine acceptance according to the socioeconomic characteristics of respondents. Using a multivariate logit regression with country fixed effects on a sample pooled across waves, we find that vaccine acceptance is more common among poorer and less well-educated individuals (Additional file 2): We find higher levels of education and higher household income quintiles to be significantly negatively correlated with vaccine acceptance. Household size and household dependency ratio, both proxies for lower household welfare outcomes, are significantly positively associated with vaccine acceptance. We also find higher vaccine acceptance among respondents who are the head of the household. Moreover, women are around five percent less likely to be willing to be vaccinated than men (Fig. 2, left panel).

There is also some variation across countries in the socioeconomic correlates of vaccine acceptance (Additional files 3 and 4). In Burkina Faso, Malawi, and Uganda, we find that respondents resident in urban areas are less likely to be willing to be vaccinated, but not in the other countries. Women are less likely to report vaccine acceptance than men in Ethiopia, Malawi, and Nigeria, while there is no difference between women and men in Uganda and Burkina Faso. Better educated individuals are less willing to be vaccinated in Nigeria and Burkina Faso, but not elsewhere.

Next, we turn to the evolution of the correlates of vaccine acceptance over time (Fig. 2 and Additional files 5 and 6). The right-hand side panel of Fig. 2 shows the coefficients of a multivariate logit regressions separately for years 2020, 2021, 2022, facilitating their comparison over time. By and large, the characteristics associated with vaccine willingness are similar across the three years even though we observe some decreases in the magnitude of the coefficients on household consumption quintiles and education over time. Conversely, the association between living in an urban area and vaccine hesitancy becomes stronger and significant only in 2022.

### Individual changing of vaccine attitudes

**Fig. 3:**
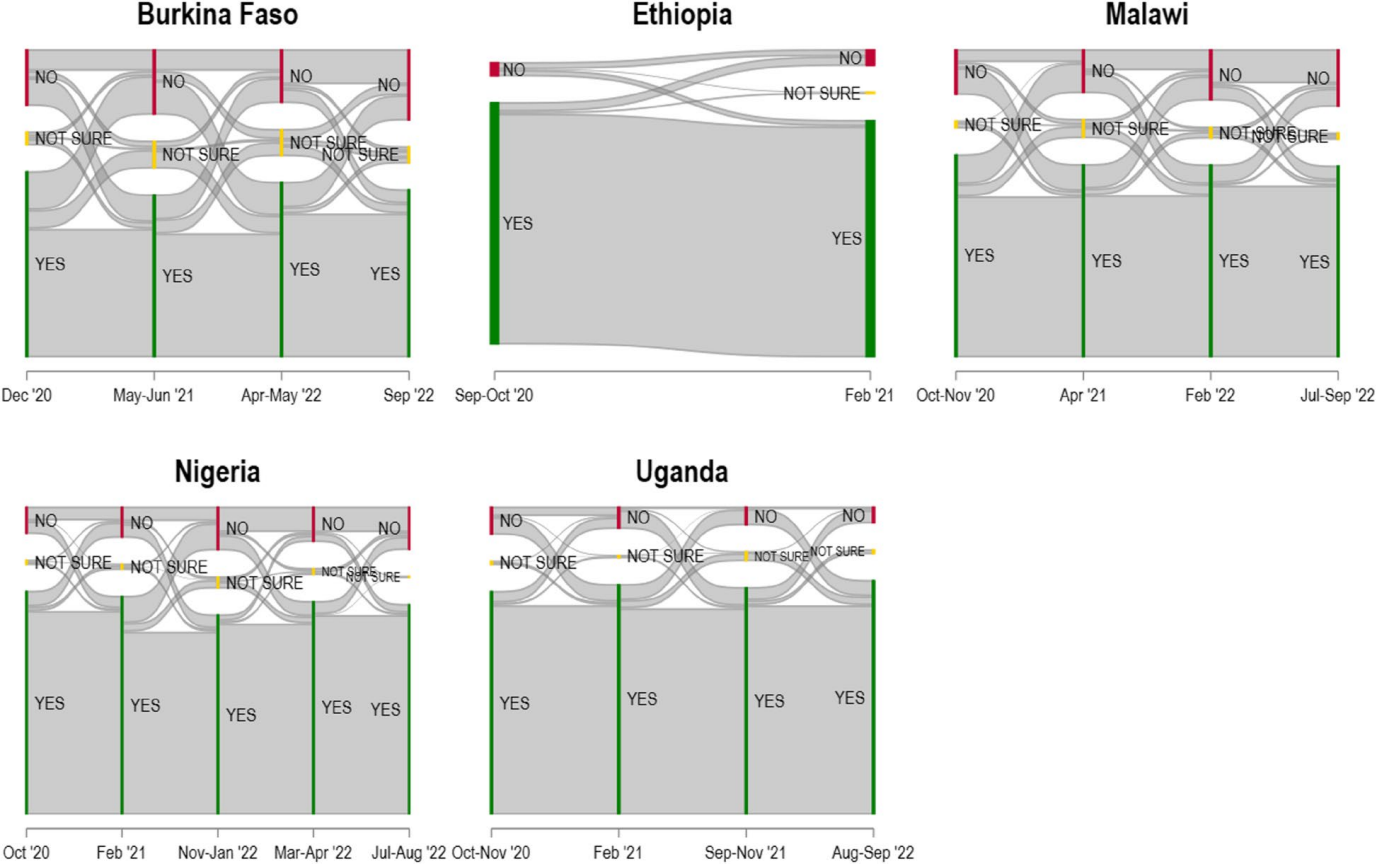
Individual-level switching of vaccine acceptance attitudes

Underlying the aggregate acceptance figures in Fig. 1, individuals are changing their vaccine attitudes through time, from accepting to hesitant and vice versa. In our data, we can track individual respondents over time and so observe attitude changes between survey rounds. Figure 3 summarizes these observations. Overall, we find that a sizeable share of respondents changed their stated attitude at least once: In Burkina Faso, this is the case for 42.8% of respondents, in Ethiopia (for which we, however, only have two rounds of data dating from 2020 and early 2021) for 6.9%, in Malawi for 38.9%, in Nigeria for 25.9%, and in Uganda for 25.7%. It is moreover evident from Fig. 3 that there is switching both from hesitant to willing and from willing to hesitance, happening between all survey rounds.

**Fig. 4:**
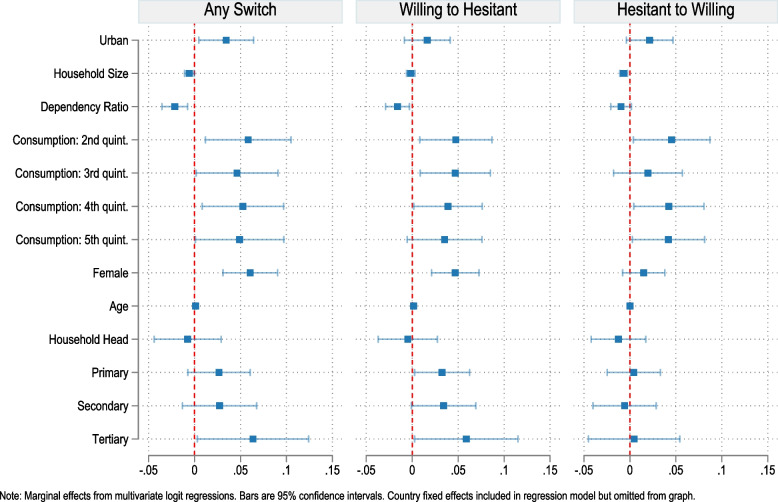
Correlates of switching COVID-19 vaccine attitudes, pooled across countries

To study the socio-economic profiles of those likely to switch vaccine attitudes, we run a series of multivariate logit regressions with country fixed effects across a pooled sample of respondents that were interviewed at least twice over the course of the COVID-19 pandemic (Fig. 4 and Additional file 7). We observe that those switching attitudes are more likely to live in richer households compared to the lowest consumption quintile and are more likely to be female, older, and highly educated. We subsequently distinguish between those respondents becoming more hesitant and those becoming more willing to get vaccinated. There are some similarities in the profiles of those who change attitudes from willing to hesitant and those who change attitudes from hesitant to willing. Attitude changes in both directions are associated with living in richer households and a lower dependency ratio of the household. But there are also some small differences: Changing attitudes toward higher hesitancy concerning COVID-19 vaccination is associated with a higher level of education, a higher age, and being female. In contrast, those becoming willing to get vaccinated over time are more likely to live in urban areas and smaller households.

### Reasons for vaccine hesitancy

**Fig. 5:**
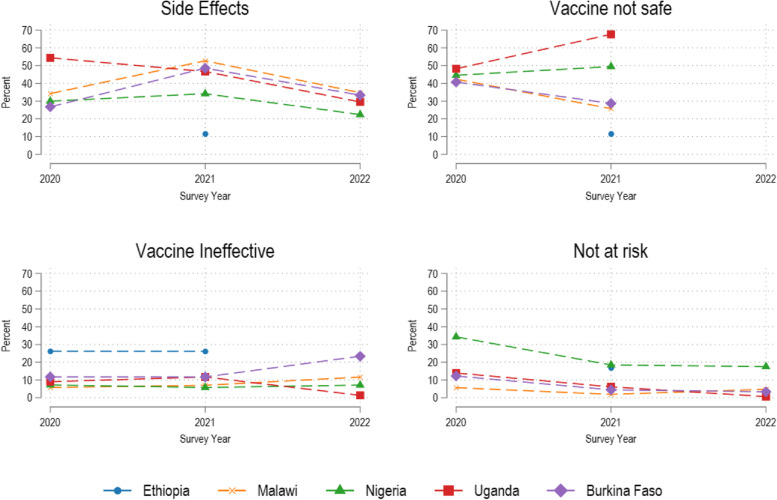
Main reasons for vaccine hesitancy over time

Overall, the most important reasons for not wanting to take a COVID-19 vaccine or being unsure of it are concerns about side effects (29.8%), concerns for vaccine safety (19.9%), and thinking that one is not at risk enough to warrant vaccination (15.1%; Additional file 8). There is some variation across countries. Concerns about side effects are much lower in Ethiopia (7.2% relative to an average of 29.8%) than in the other countries, while concerns that vaccines do not work are more common in Ethiopia and Burkina Faso than in the other countries (26.1% in Ethiopia, 18.8% in Burkina Faso relative to 8.5% overall). There is a lot of heterogeneity in hesitancy reasons over time across countries. In Nigeria, and Uganda, concerns about vaccine side effects are considerably lower in 2022 than they were in 2020. At the same time, the prevalence of respondents thinking they are not at risk enough to warrant vaccination has declined decidedly from 2020 to 2021 to 2022 (Fig. 5).

## Discussion

Using data from high frequency phone surveys, we find that rates of vaccine acceptance are high across the five countries in Sub-Saharan Africa that we study. Acceptance rates were high and 2020 and remained high in 2022. However, in three of four countries in which we have data to compare 2020 with 2022, acceptance was several percentage points lower in 2022 than in 2020, while it increased in the fourth. At the same time, COVID-19 vaccination campaigns in the region are struggling to pick up pace, as only just over one in four people have received at least two doses [16, 36]**.** The latest available vaccination rates in our study countries are 14.7% in Burkina Faso (November 27, 2022), 19.3% in Malawi (January 8, 2023), 29.3% in Nigeria (January 8, 2023), and 27.4% in Uganda (December 18, 2022) (29.8% in Ethiopia for which we do not have data from 2022) [3]. This contrasts with our findings of COVID-19 vaccine willingness ranging between 71.8% and 90.8% in these countries, suggesting that vaccine hesitancy is not a primary cause for lack of vaccine take-up.

The large gulf between declared willingness to be vaccinated and actual vaccination rates indicates that there is scope to dramatically increase vaccination rates in the region and that other factors are holding back vaccination campaigns. These include ease of access, last-mile delivery barriers, as well as vaccine supply shortages, though these appear to have eased recently – a series of recent studies has begun to explore the role these factors play in limiting take-up [7, 8, 16, 22, 37].

The discrepancy we find between high rates of vaccine acceptance and low uptake also highlights that high willingness to get vaccinated is a necessary but not sufficient condition for vaccine take-up [20]. Rather, converting high rates of vaccine acceptance into high rates of coverage is also contingent on the opportunity costs associated with getting vaccinated. Those opportunity costs are likely to be prohibitively high for many living in Sub-Saharan Africa in light of the structural barriers to access vaccines that the literature has identified [7, 8, 16, 22, 37]. Furthermore, the decision to get vaccinated, as opposed to willingness to get vaccinated in principle, is influenced by the perceived need for or urgency of vaccination [38]. As the salience of the pandemic in Sub-Saharan African countries has been low in comparison to their high-income counterparts [39], this may have created the impression that COVID-19 has been somewhat less deleterious in the region and lowered the perceived risk of falling severely ill. Together with competing priorities such as the struggle to make ends meet or domestic care responsibilities, these factors may contribute to the gap between stated vaccine acceptance and actual uptake.

While not unique to the COVID-19 pandemic, the trade-off between opportunity costs of vaccination and perceived benefits may be somewhat more acute in the current case. This is due to the relative novelty of the disease, the short timeframe over which vaccines were rolled out, concurrent livelihoods crisis, and the fact that vaccination campaigns target the whole population as opposed to primarily children. Even though vaccine hesitancy is complex and context-specific [40], we argue that these findings carry some significance for future vaccination efforts. Specifically, they demonstrate the importance of addressing demand and supply side constraints in concert in order to achieve high rates of coverage and that vaccine hesitancy can hardly be considered a binding constraint in the absence of adequate and accessible supply [21, 24, 30].

Despite high acceptance rates, our analysis finds that vaccine attitudes can and do change over time in a significant share of the population. For vaccine campaigns, this means that efforts to resolve misinformation and address concerns regarding the safety and effectiveness of vaccines among hesitant population groups need to be ongoing and effectively targeted. Lastly, our findings also highlight that vaccine hesitancy is a country-specific phenomenon with at times substantial variation in vaccine attitudes and their dynamics, correlates of hesitancy and hesitancy reasons. Any policy recommendations thus need to take into account the specific country context.

Our study faces the challenges and limitations of phone survey data collection on vaccination. There is some sample selection at the household level due to under-coverage, non-response, and attrition, whose potential impacts are, however, attenuated by our recalibrated sampling weights [34]. The purposive selection of respondents leads to the over-representation of certain population groups and the under-representation of others [35]. Survey data relies on respondent declarations, a method that is vulnerable to respondents’ incentives, misreporting, and misperceptions. Finally, our estimates cover five countries with a population of around 433 million, but they need not be representative the entire region.

## Conclusions

We find overall high rates vaccine acceptance across our study countries and over a 2-year time horizon, never dipping below 68%. Yet, we notice a decline of several percentage points in the willingness to be vaccinated in three countries from 2020 to 2022, while in one country vaccine acceptance increases. Overall, women, better educated individuals and those living in better-off households are more likely to express vaccine hesitancy. Vaccine hesitancy is lower in larger households and among heads of the household. Underlying vaccine acceptance and its correlates, there are important country differences. The main reasons for vaccine hesitancy are concerns about side effects and vaccine safety as well as perceptions around COVID-19 risk.

While this is not the only study analyzing COVID-19 vaccine attitudes in Sub-Saharan Africa, there has been lack of longitudinal evidence to assess dynamics over time. This study fills this gap by using panel survey data collected in high frequency phone surveys over the span of almost two years.

### Supplementary Information

**Additional file 1: Table A. 1.** Vaccine acceptance over time.

**Additional file 2: Table A. 2.** Correlates of vaccine acceptance.

**Additional file 3: Table A. 3.** Correlates of vaccine acceptance by country.

**Additional file 4: Figure A. 1.** Correlates of vaccine acceptance by country, pooled across time.

**Additional file 5: Figure A. 2.** Correlates of vaccine acceptance by year.

**Additional file 6: Table A. 4.** Correlates of vaccine acceptance by year.

**Additional file 7: Table A. 5.** Correlates of switching vaccine attitudes, pooled across countries.

**Additional file 8: Table A. 6.** Reasons for vaccine hesitancy.

## Data Availability

The raw data used in this study as well as the full questionnaires have been made publicly available through the World Bank’s microdata library: • Burkina Faso (https://microdata.worldbank.org/index.php/catalog/3768) • Ethiopia (https://microdata.worldbank.org/index.php/catalog/3716) • Malawi (https://microdata.worldbank.org/index.php/catalog/3766) • Nigeria (https://microdata.worldbank.org/index.php/catalog/4444) • Uganda (https://microdata.worldbank.org/index.php/catalog/3765)
