# Supplementary material for: The evolution of COVID-19 vaccine hesitancy in Sub-Saharan Africa: evidence from panel survey data
Source: BMC Proc. 2023 Jul 6;17(Suppl 7):8. doi: 10.1186/s12919-023-00266-x (PMC10324117; doi:10.1186/s12919-023-00266-x)
Supplement: Supplementary file 1 — Additional file 1: Table A. 1. Vaccine acceptance over time. [file 12919_2023_266_MOESM1_ESM.docx]

## Additional File 1

Table A. 1. Vaccine Acceptance Over Time

|  | | | | | | |
| --- | --- | --- | --- | --- | --- | --- |
| **Survey Period** | Ethiopia | Malawi | Nigeria | Uganda | Burkina Faso |  |
| Sep-Dec 2020 | 97.9 | 82.7 | 86.2 | 84.3 | 79.5 |  |
|  | (97.2 to 98.6) | (80.0 to 85.4) | (83.9 to 88.5) | (82.0 to 86.5) | (76.9 to 82.1) |  |
| Feb-Jun 2021 | 96.5 | 73.6 | 83.4 | 88.4 | 68.2 |  |
|  | (95.4 to 97.7) | (70.3 to 77.0) | (80.7 to 86.2) | (86.4 to 90.5) | (63.9 to 72.5) |  |
| Sep '21 - Feb 2022 |  | 75.1 | 78.4 | 88.1 |  |  |
|  |  | (71.6 to 78.7) | (76.1 to 80.6) | (86.0 to 90.3) |  |  |
| Mar-May 2022 |  |  | 84.0 |  | 74.4 |  |
|  |  |  | (82.0 to 86.0) |  | (71.5 to 77.2) |  |
| Jun-Sep 2022 |  | 79.9 | 83.2 | 90.8 | 71.8 |  |
|  |  | (76.4 to 83.4) | (81.1 to 85.4) | (88.9 to 92.8) | (68.6 to 74.9) |  |
| *N (Sep-Dec 2020)* | 2,704 | 1,589 | 1,762 | 2,135 | 1,944 |  |
| *N (Feb-Jun 2021)* | 2,178 | 1,517 | 1,700 | 2,121 | 1,030 |  |
| *N (Sep '21 - Feb 2022)* |  | 1,449 | 2,983 | 1,885 |  |  |
| *N (Mar-May 2022)* |  |  | 2,661 |  | 1,847 |  |
| *N (Jun-Sep 2022)* |  | 1,348 | 2,575 | 1,872 | 1,707 |  |
| Note: Vaccine acceptance over time. Survey dates: Burkina Faso (Dec '20, May-Jun '21, Apr-May '22, Aug-Sep '22), Ethiopia (Sep-Oct '20, Feb '21), Malawi (Oct-Nov '20, Apr '21, Feb '22, Jul-Sep '22), Nigeria (Oct '20, Feb '21, Dec'21-Jan'22, Mar-Apr '22, Jul-Sep '22), Uganda (Oct-Nov '20, Feb '21, Sep-Nov '21, Aug-Sep '22). 95% confidence intervals in parentheses. | | | | | |  |
