# Supplementary material for: The evolution of COVID-19 vaccine hesitancy in Sub-Saharan Africa: evidence from panel survey data
Source: BMC Proc. 2023 Jul 6;17(Suppl 7):8. doi: 10.1186/s12919-023-00266-x (PMC10324117; doi:10.1186/s12919-023-00266-x)
Supplement: Supplementary file 2 — Additional file 2: Table A. 2. Correlates of vaccine acceptance. [file 12919_2023_266_MOESM2_ESM.docx]

## Additional File 2

Table A. 2. Correlates of vaccine acceptance

| **Correlates of Vaccine Acceptance** | |
| --- | --- |
|  | (1) |
| VARIABLES | Pooled |
|  |  |
| Urban | -0.0103 |
|  | (0.00704) |
| Household Size | 0.00732*** |
|  | (0.00147) |
| Dependency Ratio | 0.0134*** |
|  | (0.00407) |
| Consumption quintile = 2, Consumption: 2nd quint. | -0.0234** |
|  | (0.0112) |
| Consumption quintile = 3, Consumption: 3rd quint. | -0.0474*** |
|  | (0.0109) |
| Consumption quintile = 4, Consumption: 4th quint. | -0.0499*** |
|  | (0.0111) |
| Consumption quintile = 5, Consumption: 5th quint. | -0.0586*** |
|  | (0.0120) |
| Female | -0.0548*** |
|  | (0.00767) |
| Age | -0.000345 |
|  | (0.000249) |
| Household Head | 0.0212** |
|  | (0.00923) |
| Highest education completed = 1, Primary | -0.0180** |
|  | (0.00875) |
| Highest education completed = 2, Secondary | -0.0413*** |
|  | (0.00963) |
| Highest education completed = 3, Tertiary | -0.0574*** |
|  | (0.0140) |
|  |  |
| Observations | 37,408 |
| Country FE | YES |
| Pseudo R2 | 0.0899 |
| Note: Marginal effects from multivariate logistic regression, pooled across countries and survey rounds. Malawi only has quintiles of a household wealth index instead of total household consumption. Standard errors in parentheses. *** p<0.01, ** p<0.05, * p<0.1 | |
