# Supplementary material for: The evolution of COVID-19 vaccine hesitancy in Sub-Saharan Africa: evidence from panel survey data
Source: BMC Proc. 2023 Jul 6;17(Suppl 7):8. doi: 10.1186/s12919-023-00266-x (PMC10324117; doi:10.1186/s12919-023-00266-x)
Supplement: Supplementary file 3 — Additional file 3: Table A. 3. Correlates of vaccine acceptance by country. [file 12919_2023_266_MOESM3_ESM.docx]

## Additional File 3

Table A. 3. Correlates of vaccine acceptance by country

| **Correlates of Vaccine Acceptance (By Country)** | | | | | |
| --- | --- | --- | --- | --- | --- |
|  | (1) | (2) | (3) | (4) | (5) |
| VARIABLES | Ethiopia | Malawi | Nigeria | Uganda | Burkina Faso |
|  |  |  |  |  |  |
| Urban | -0.00886 | -0.0430** | -0.00108 | -0.0461*** | -0.128*** |
|  | (0.00838) | (0.0181) | (0.0111) | (0.0123) | (0.0168) |
| Household Size | 0.00527*** | 0.00695* | 0.00832*** | 0.00433** | 0.00442 |
|  | (0.00163) | (0.00373) | (0.00235) | (0.00209) | (0.00279) |
| Dependency Ratio | 0.000656 | 0.0126 | 0.0192*** | -0.00573 | 0.00368 |
|  | (0.00521) | (0.00866) | (0.00671) | (0.00591) | (0.0105) |
| Consumption quintile = 2, Consumption: 2nd quint. | -0.00142 | -0.0336 | -0.0281 | -0.0162 | 0.0263 |
|  | (0.00845) | (0.0313) | (0.0184) | (0.0171) | (0.0287) |
| Consumption quintile = 3, Consumption: 3rd quint. | -0.0231** | -0.107*** | -0.0381** | -0.0621*** | -0.0236 |
|  | (0.0109) | (0.0289) | (0.0183) | (0.0179) | (0.0285) |
| Consumption quintile = 4, Consumption: 4th quint. | -0.00634 | -0.104*** | -0.0447** | -0.0555*** | -0.0414 |
|  | (0.00790) | (0.0282) | (0.0191) | (0.0171) | (0.0297) |
| Consumption quintile = 5, Consumption: 5th quint. | -0.0403** | -0.0623** | -0.0576*** | -0.0481*** | -0.0794** |
|  | (0.0157) | (0.0302) | (0.0205) | (0.0185) | (0.0339) |
| Female | -0.0280*** | -0.0644*** | -0.0753*** | 0.00398 | 0.0164 |
|  | (0.00862) | (0.0184) | (0.0125) | (0.0127) | (0.0225) |
| Age | -7.20e-05 | 0.00254*** | -0.000886** | -0.000457 | 0.000394 |
|  | (0.000183) | (0.000642) | (0.000419) | (0.000380) | (0.000641) |
| Household Head | 0.0145* | 0.00448 | 0.0336** | -0.00338 | 0.0229 |
|  | (0.00873) | (0.0210) | (0.0150) | (0.0145) | (0.0261) |
| Highest education completed = 1, Primary | 0.00207 | -0.00318 | -0.0321** | 0.0137 | -0.00809 |
|  | (0.00805) | (0.0173) | (0.0156) | (0.0125) | (0.0229) |
| Highest education completed = 2, Secondary | -0.0217 | -0.0264 | -0.0555*** | 0.0152 | -0.0957*** |
|  | (0.0154) | (0.0241) | (0.0149) | (0.0166) | (0.0284) |
| Highest education completed = 3, Tertiary | -0.00301 | -0.0367 | -0.0865*** | 0.0132 | -0.0973** |
|  | (0.00986) | (0.0405) | (0.0204) | (0.0259) | (0.0493) |
|  |  |  |  |  |  |
| Observations | 4,807 | 7,399 | 11,065 | 7,777 | 6,360 |
| Survey Wave FE | YES | YES | YES | YES | YES |
| Pseudo R2 | 0.126 | 0.0710 | 0.0608 | 0.0266 | 0.0738 |
| Note: Marginal effects from multivariate logistic regression, by country. Malawi only has quintiles of a household wealth index instead of total household consumption. Standard errors in parentheses. *** p<0.01, ** p<0.05, * p<0.1 | | | | | |
