# Supplementary figures and images for: The evolution of COVID-19 vaccine hesitancy in Sub-Saharan Africa: evidence from panel survey data
Source: BMC Proc. 2023 Jul 6;17(Suppl 7):8. doi: 10.1186/s12919-023-00266-x (PMC10324117; doi:10.1186/s12919-023-00266-x)

## Additional File 4

Figure A. 1. Correlates of vaccine acceptance by country, pooled across time


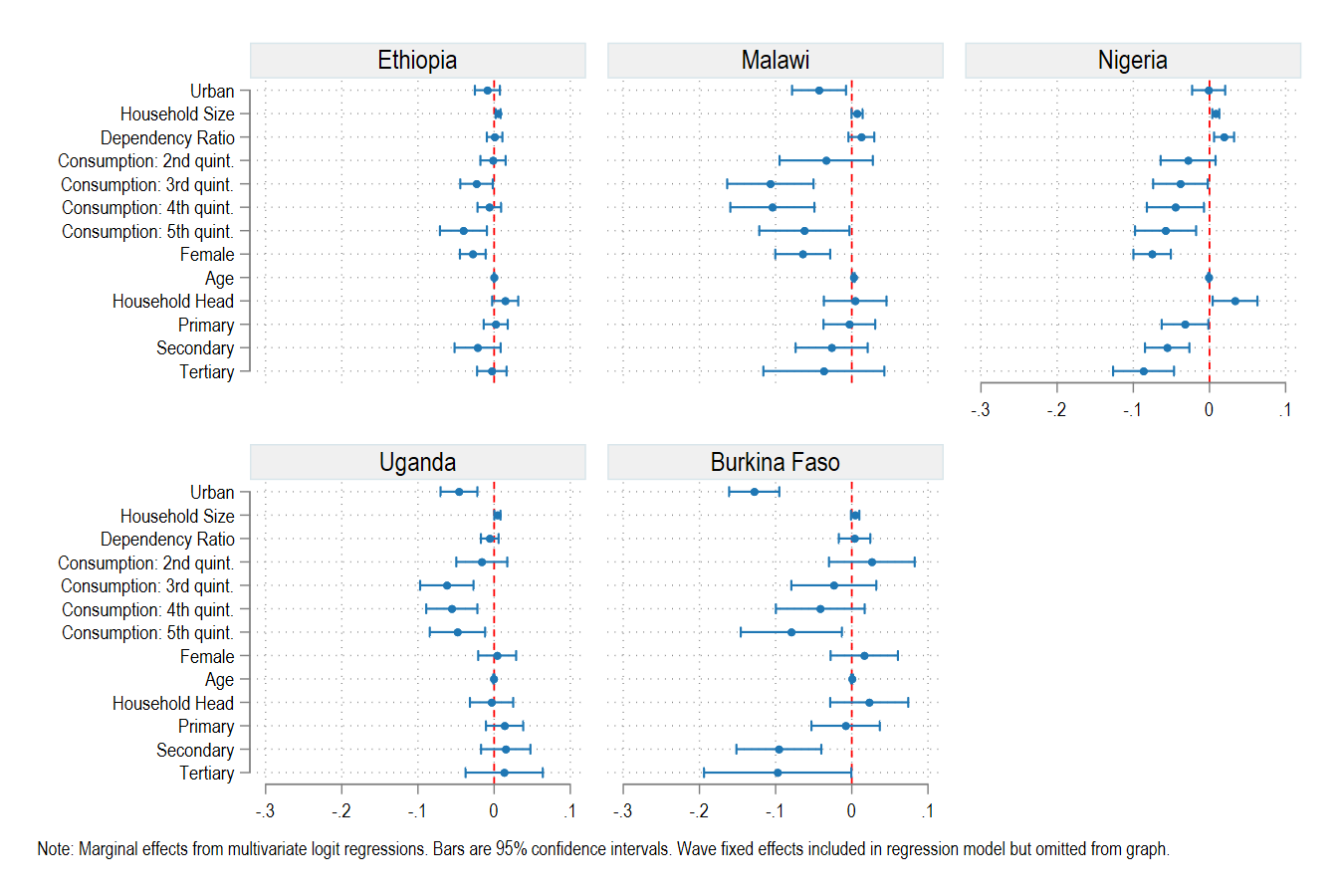

Supplement: Supplementary file 4 — Additional file 4: Figure A. 1. Correlates of vaccine acceptance by country, pooled across time. [file 12919_2023_266_MOESM4_ESM.docx]

## Additional File 5

Figure A. 2. Correlates of vaccine acceptance by year
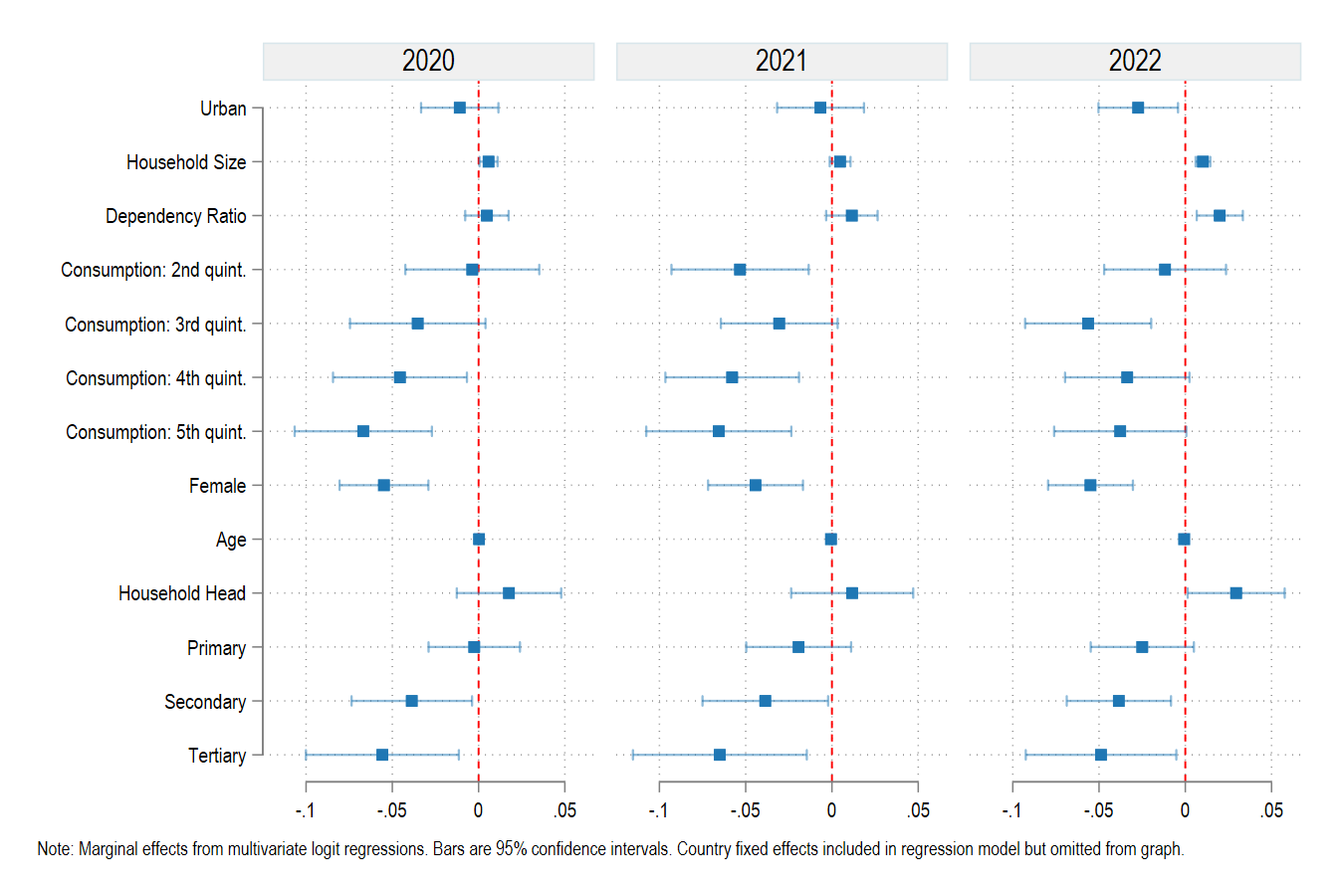

Supplement: Supplementary file 5 — Additional file 5: Figure A. 2. Correlates of vaccine acceptance by year. [file 12919_2023_266_MOESM5_ESM.docx]
