# Supplementary material for: The evolution of COVID-19 vaccine hesitancy in Sub-Saharan Africa: evidence from panel survey data
Source: BMC Proc. 2023 Jul 6;17(Suppl 7):8. doi: 10.1186/s12919-023-00266-x (PMC10324117; doi:10.1186/s12919-023-00266-x)
Supplement: Supplementary file 6 — Additional file 6: Table A. 4. Correlates of vaccine acceptance by year. [file 12919_2023_266_MOESM6_ESM.docx]

## Additional File 6

Table A. 4. Correlates of vaccine acceptance by year

| **Correlates of Vaccine Acceptance** | | | |
| --- | --- | --- | --- |
|  | (1) | (2) | (3) |
| VARIABLES | 2020 | 2021 | 2022 |
|  |  |  |  |
| Urban | -0.0109 | -0.00662 | -0.0273** |
|  | (0.0114) | (0.0128) | (0.0118) |
| Household Size | 0.00578** | 0.00472 | 0.0102*** |
|  | (0.00266) | (0.00308) | (0.00218) |
| Dependency Ratio | 0.00480 | 0.0115 | 0.0200*** |
|  | (0.00642) | (0.00761) | (0.00681) |
| Consumption quintile = 2, Consumption: 2nd quint. | -0.00365 | -0.0533*** | -0.0117 |
|  | (0.0198) | (0.0203) | (0.0180) |
| Consumption quintile = 3, Consumption: 3rd quint. | -0.0353* | -0.0306* | -0.0563*** |
|  | (0.0200) | (0.0173) | (0.0186) |
| Consumption quintile = 4, Consumption: 4th quint. | -0.0456** | -0.0578*** | -0.0337* |
|  | (0.0198) | (0.0198) | (0.0184) |
| Consumption quintile = 5, Consumption: 5th quint. | -0.0668*** | -0.0656*** | -0.0377* |
|  | (0.0203) | (0.0215) | (0.0196) |
| Female | -0.0549*** | -0.0443*** | -0.0549*** |
|  | (0.0131) | (0.0140) | (0.0125) |
| Age | 0.000287 | -0.000558 | -0.000633 |
|  | (0.000363) | (0.000465) | (0.000419) |
| Household Head | 0.0176 | 0.0117 | 0.0295** |
|  | (0.0154) | (0.0180) | (0.0143) |
| Highest education completed = 1, Primary | -0.00251 | -0.0193 | -0.0249 |
|  | (0.0135) | (0.0155) | (0.0153) |
| Highest education completed = 2, Secondary | -0.0387** | -0.0386** | -0.0385** |
|  | (0.0178) | (0.0186) | (0.0154) |
| Highest education completed = 3, Tertiary | -0.0558** | -0.0650** | -0.0489** |
|  | (0.0226) | (0.0257) | (0.0223) |
| Country = 2, Malawi | -0.140*** | -0.217*** |  |
|  | (0.0141) | (0.0195) |  |
| Country = 3, Nigeria | -0.114*** | -0.129*** | 0.0497*** |
|  | (0.0137) | (0.0157) | (0.0154) |
| Country = 4, Uganda | -0.117*** | -0.0697*** | 0.147*** |
|  | (0.0122) | (0.0109) | (0.0158) |
| Country = 5, Burkina Faso | -0.234*** | -0.311*** | -0.0817*** |
|  | (0.0195) | (0.0286) | (0.0190) |
|  |  |  |  |
| Observations | 9,949 | 10,206 | 15,704 |
| Survey Wave FE | YES | YES | YES |
| Pseudo R2 | 0.128 | 0.101 | 0.0530 |
| Note: Marginal effects from multivariate logistic regression, by year. Standard errors in parentheses. *** p<0.01, ** p<0.05, * p<0.1 | | | |
