# Supplementary material for: The evolution of COVID-19 vaccine hesitancy in Sub-Saharan Africa: evidence from panel survey data
Source: BMC Proc. 2023 Jul 6;17(Suppl 7):8. doi: 10.1186/s12919-023-00266-x (PMC10324117; doi:10.1186/s12919-023-00266-x)
Supplement: Supplementary file 7 — Additional file 7: Table A. 5. Correlates of switching vaccine attitudes, pooled across countries. [file 12919_2023_266_MOESM7_ESM.docx]

## Additional File 7

Table A 5: Correlates of switching vaccine attitudes, pooled across countries.

| **Correlates of switching vaccine attitudes** | | | |
| --- | --- | --- | --- |
|  | (1) | (2) | (3) |
| VARIABLES | Any Switch | Willing to Hesitant | Hesitant to Willing |
|  |  |  |  |
| Urban | 0.0344** | 0.0164 | 0.0215* |
|  | (0.0152) | (0.0127) | (0.0129) |
| Household Size | -0.00568** | -0.00165 | -0.00680*** |
|  | (0.00267) | (0.00233) | (0.00214) |
| Dependency Ratio | -0.0215*** | -0.0160** | -0.00970* |
|  | (0.00713) | (0.00663) | (0.00571) |
| Consumption quintile = 2, Consumption: 2nd quint. | 0.0584** | 0.0475** | 0.0454** |
|  | (0.0237) | (0.0201) | (0.0213) |
| Consumption quintile = 3, Consumption: 3rd quint. | 0.0462** | 0.0469** | 0.0196 |
|  | (0.0227) | (0.0194) | (0.0191) |
| Consumption quintile = 4, Consumption: 4th quint. | 0.0526** | 0.0390** | 0.0423** |
|  | (0.0226) | (0.0190) | (0.0195) |
| Consumption quintile = 5, Consumption: 5th quint. | 0.0491** | 0.0352* | 0.0419** |
|  | (0.0246) | (0.0208) | (0.0201) |
| Female | 0.0606*** | 0.0468*** | 0.0150 |
|  | (0.0152) | (0.0132) | (0.0117) |
| Age | 0.00114** | 0.00146*** | 3.57e-05 |
|  | (0.000465) | (0.000424) | (0.000379) |
| Household Head | -0.00748 | -0.00481 | -0.0124 |
|  | (0.0186) | (0.0164) | (0.0151) |
| Highest education completed = 1, Primary | 0.0267 | 0.0325** | 0.00421 |
|  | (0.0173) | (0.0153) | (0.0148) |
| Highest education completed = 2, Secondary | 0.0272 | 0.0341* | -0.00581 |
|  | (0.0206) | (0.0179) | (0.0175) |
| Highest education completed = 3, Tertiary | 0.0636** | 0.0588** | 0.00462 |
|  | (0.0309) | (0.0287) | (0.0254) |
|  |  |  |  |
| Observations | 10,750 | 10,750 | 10,750 |
| Country FE | YES | YES | YES |
| Pseudo R2 | 0.115 | 0.0950 | 0.108 |
| Note: Marginal effects from multivariate logistic regression, pooled across countries and survey rounds. Standard errors in parentheses. *** p<0.01, ** p<0.05, * p<0.1 | | | |
