# Supplementary material for: The evolution of COVID-19 vaccine hesitancy in Sub-Saharan Africa: evidence from panel survey data
Source: BMC Proc. 2023 Jul 6;17(Suppl 7):8. doi: 10.1186/s12919-023-00266-x (PMC10324117; doi:10.1186/s12919-023-00266-x)
Supplement: Supplementary file 8 — Additional file 8: Table A. 6. Reasons for vaccine hesitancy. [file 12919_2023_266_MOESM8_ESM.docx]

## Additional File 8

Table A. 6. Reasons for vaccine hesitancy

| **Reasons for Vaccine Hesitancy by Country** | | | | | | |
| --- | --- | --- | --- | --- | --- | --- |
| **Hesitancy Reason** | Ethiopia | Malawi | Nigeria | Uganda | Burkina Faso | Pooled |
| Vaccine does not work | 26.1 | 7.6 | 6.8 | 7.8 | 18.8 | 8.5 |
|  | (14.2 to 38.1) | (5.8 to 9.3) | (5.2 to 8.5) | (5.2 to 10.5) | (16.0 to 21.6) | (7.3 to 9.8) |
| Vaccine is not safe | 35.7 | 23.7 | 16.9 | 32.0 | 14.0 | 19.9 |
|  | (24.6 to 46.8) | (21.0 to 26.4) | (13.9 to 19.9) | (27.6 to 36.4) | (11.7 to 16.4) | (17.9 to 22.0) |
| Concerned about sideeffects | 7.2 | 40.2 | 25.9 | 42.0 | 34.3 | 29.8 |
|  | (1.5 to 12.8) | (37.0 to 43.3) | (22.9 to 29.0) | (37.4 to 46.6) | (31.0 to 37.6) | (27.7 to 31.9) |
| Not enough at risk | 10.4 | 4.0 | 20.4 | 7.1 | 5.6 | 15.1 |
|  | (2.9 to 17.9) | (2.7 to 5.2) | (17.6 to 23.2) | (4.7 to 9.4) | (3.8 to 7.3) | (13.3 to 17.0) |
| Do not trust vaccines in general | 2.6 | 12.0 | 9.3 | 15.7 | 4.3 | 9.8 |
|  | (0.3 to 4.9) | (9.8 to 14.2) | (7.3 to 11.3) | (12.3 to 19.1) | (3.0 to 5.6) | (8.4 to 11.2) |
| Religious reasons | 22.3 | 2.3 | 3.1 | 0.5 | 0.1 | 3.0 |
|  | (10.9 to 33.8) | (1.2 to 3.3) | (2.1 to 4.0) | (0.0 to 1.0) | (0.0 to 0.4) | (2.3 to 3.8) |
| Had COVID already |  | 0.0 | 0.4 | 0.5 | 0.1 | 0.3 |
|  |  | (0.0 to 0.0) | (0.0 to 0.8) | (0.0 to 1.2) | (0.0 to 0.2) | (0.1 to 0.6) |
| Would get infected at vax site |  | 0.0 | 0.6 | 0.4 | 0.2 | 0.4 |
|  |  | (0.0 to 0.1) | (0.2 to 0.9) | (0.0 to 0.7) | (0.0 to 0.4) | (0.2 to 0.7) |
| Vaccination site too far away |  | 0.5 | 1.1 | 1.2 | 0.2 | 0.9 |
|  |  | (0.0 to 1.0) | (0.3 to 1.9) | (0.1 to 2.4) | (0.0 to 0.5) | (0.4 to 1.5) |
| No time to get vaccinated |  | 0.5 | 1.1 | 0.8 | 0.7 | 0.9 |
|  |  | (0.0 to 1.2) | (0.4 to 1.8) | (0.0 to 1.5) | (0.2 to 1.1) | (0.4 to 1.4) |
| Vaccines available are inferior |  | 0.3 | 2.0 | 0.2 | 5.3 | 1.8 |
|  |  | (0.0 to 0.7) | (1.3 to 2.8) | (0.0 to 0.6) | (3.9 to 6.6) | (1.3 to 2.3) |
| Wouldn't get preferred vaccine |  | 1.4 | 1.6 | 0.4 | 0.9 | 1.3 |
|  |  | (0.7 to 2.1) | (0.8 to 2.4) | (0.0 to 1.0) | (0.4 to 1.4) | (0.8 to 1.8) |
| Not a priority |  | 8.5 | 9.3 | 2.6 | 9.6 | 8.1 |
|  |  | (6.4 to 10.5) | (7.4 to 11.2) | (1.2 to 4.0) | (7.5 to 11.7) | (6.9 to 9.4) |
| COVID does not exist here |  | 0.4 | 9.5 | 0.4 | 4.5 | 6.5 |
|  |  | (0.0 to 0.8) | (7.6 to 11.4) | (0.0 to 1.2) | (3.1 to 5.9) | (5.3 to 7.7) |
| Medical reasons |  | 0.4 | 2.2 | 3.4 | 0.3 | 1.9 |
|  |  | (0.0 to 0.8) | (1.3 to 3.0) | (1.6 to 5.3) | (0.1 to 0.5) | (1.3 to 2.5) |
| Distrust the government |  |  | 2.4 |  | 4.5 | 1.9 |
|  |  |  | (1.6 to 3.3) |  | (3.1 to 5.9) | (1.3 to 2.4) |
| Other | 4.1 | 1.2 | 12.6 | 4.7 | 3.8 | 9.2 |
|  | (0.0 to 8.2) | (0.5 to 1.8) | (10.6 to 14.7) | (2.7 to 6.8) | (2.7 to 5.0) | (7.8 to 10.5) |
| *N* | 307 | 2,170 | 2,040 | 887 | 1,883 | 7,224 |
| Note: Reasons for vaccine hesitancy by country. All values in percent. 95% confidence intervals in parentheses. | | | | | | |
